# Spatial‐hindrance‐based pro‐Adalimumab prevents anti‐idiotypic antibody interference in pharmacokinetic and therapeutic efficacy

**DOI:** 10.1002/btm2.70015

**Published:** 2025-04-01

**Authors:** Bo‐Cheng Huang, Yu‐Tung Chen, Yun‐Chi Lu, Kai‐Wen Ho, Shih‐Ting Hong, Tzu‐Yi Liao, I‐Hsuan Wu, En‐Shuo Liu, Jun‐Min Liao, Fang‐Ming Chen, Chia‐Ching Li, Chih‐Hung Chuang, Chiao‐Yun Chen, Tian‐Lu Cheng

**Affiliations:** ^1^ Drug Development and Value Creation Research Center Kaohsiung Medical University Kaohsiung Taiwan; ^2^ Department of Surgery Faculty of Medicine College of Medicine Kaohsiung Medical University Kaohsiung Taiwan; ^3^ Graduate Institute of Medicine, College of Medicine Kaohsiung Medical University Kaohsiung Taiwan; ^4^ PrecisemAb Biotech Co., Ltd Kaohsiung Taiwan; ^5^ Center for Biomarkers and Biotech Drugs Kaohsiung Medical University Kaohsiung Taiwan; ^6^ Department of Surgery Kaohsiung Municipal Ta‐Tung Hospital Kaohsiung Taiwan; ^7^ Division of Breast Oncology And Surgery, Department of Surgery Kaohsiung Medical University Chung‐Ho Memorial Hospital Kaohsiung Taiwan; ^8^ Department of Medical Laboratory Science and Biotechnology Kaohsiung Medical University Kaohsiung Taiwan; ^9^ Department of Medical Imaging Kaohsiung Medical University Hospital Kaohsiung Taiwan; ^10^ School of Post‐Baccalaureate Medicine, College of Medicine Kaohsiung Medical University Kaohsiung Taiwan; ^11^ Department of Biomedical Science and Environmental Biology Kaohsiung Medical University Kaohsiung Taiwan

**Keywords:** Ab lock (hinge), avoid interference from anti‐id Abs, pro‐adalimumab, protease activation, rheumatoid arthritis

## Abstract

Adalimumab (Humira) represents a major advance in rheumatoid arthritis (RA) therapy. However, with long‐term administration of Adalimumab, anti‐idiotypic antibody (anti‐Id Ab) accelerates the Adalimumab clearance rate and reduces the therapeutic effect. To avoid the interference of anti‐Id Ab, we used an autologous hinge region as a spatial‐hindrance‐based Ab lock and connected it to the N‐terminal of the light chain and heavy chain via substrate peptides (MMP‐2/9) to cover the CDR binding site of Adalimumab to generate pro‐Adalimumab. The Ab lock masks the complementarity‐determining regions (CDRs) of Adalimumab, thus avoiding interference from anti‐Id Ab. Pro‐Adalimumab demonstrated a 241.6 times weaker binding ability to TNFɑ than Adalimumab. In addition, pro‐Adalimumab showed a 46.6‐fold greater blocking of anti‐Adalimumab Id Ab in comparison to Adalimumab prior to activation. Similar results were observed with other clinical antibodies, such as pro‐Infliximab (anti‐TNFɑ Ab) and pro‐Nivolumab (anti‐PD‐1). Furthermore, pro‐Adalimumab maintained consistent pharmacokinetics regardless of the presence of anti‐Adalimumab Id antibodies, while Adalimumab showed a 49% clearance increase, resulting in a near complete loss of function. Additionally, pro‐Adalimumab was able to avoid neutralization and efficiently reduce RA progression in the presence of anti‐Adalimumab Id Ab in vivo. In summary, we developed a pro‐Adalimumab that avoids interference from anti‐Id Abs, thereby addressing the biggest issue limiting clinical efficacy. The findings enclosed herein may have potentially broad application in antibody therapies.

**Translational Impact Statement**

Our results provide strong evidence that the pro‐Adalimumab possesses important advantages: 1. Pro‐Adalimumab can mitigate anti‐Id Ab interference and extend drug half‐life. 2. Pro‐Adalimumab can serve as a second‐line drug for anti‐Id Ab‐mediated therapy failure. 3. The spatial‐hindrance‐based Ab‐lock can convert any antibody into prodrug forms. By simply genetic synthesis and recombination, the hinge region can be connected to the N terminal of the antibody via protease substrates, resulting in pro‐antibody that any laboratory can construct and produce.

## 1 BACKGROUND

Monoclonal antibody (Ab)‐based therapy has become one of the most successful strategies for treating autoimmune diseases,^1^, ^2^, ^3^, ^4^ infectious diseases,^5^, ^6^ and malignancies^7^, ^8^, ^9^, ^10^ based on its high specificity to target antigens. However, numerous FDA‐approved Ab drugs induce the production of anti‐drug Abs in patients during treatment,^11^ especially anti‐idiotypic Abs (anti‐Id Abs) due to the variation of the complementarity‐determining region (CDR) loop of the antigen‐binding site of Ab.^12^ Anti‐Id Abs can neutralize the activity of an Ab drug by binding to the CDR loop, thereby accelerating its clearance, resulting in treatment failure and even increasing the frequency of immune‐mediated adverse effects.^13^, ^14^, ^15^, ^16^, ^17^ Humira® (Adalimumab), the world's best‐selling drug, constituted a major advance in rheumatoid arthritis (RA) therapy by neutralizing tumor necrosis factor‐alpha (TNFɑ) in the disease region.^18^, ^19^ However, it is reported that patients given Adalimumab may elicit an anti‐Adalimumab Ab, which decreases its efficiency. For example, Bartelds and colleagues suggested that 28% of Adalimumab‐treated RA patients will induce anti‐Adalimumab Ab within 3 years of treatment.^20^ Menting and colleagues also observed that an anti‐Adalimumab Ab was generated in 49% of Adalimumab‐treated patients during one‐year treatment, leading to 5–10‐fold lower serum concentration of Adalimumab and often no response to the treatment as compared with the patients without anti‐Adalimumab Ab.^21^ Thus, developing a strategy that can inhibit the interference effect of anti‐Id Ab and enhance the therapeutic efficacy of Ab drugs during disease treatment is an important unmet need.

Several strategies have been developed to prevent interference from anti‐Id Abs during disease treatment with Ab drugs. Two approaches for reducing the immunogenicity of antibody drugs involve using concomitant immunosuppressive medications to inhibit the secretion of anti‐Id Ab from immune cells or utilizing biosimilar versions of antibody drugs.^22^ One strategy involved combining Ab drugs with an immunomodulator to induce tolerogenic immune cells. Krieckart et al. found that co‐medication with methotrexate (MTX)^23^ and Adalimumab to treat RA could cause 4‐fold fewer anti‐Adalimumab Ab‐positive patients compared to treatment with Adalimumab monotherapy.^24^ Burmester and colleagues also demonstrated that increasing doses of MTX could improve the concentration of Adalimumab by 1.5‐fold and also clinical outcomes of RA patients in a dose‐dependent manner.^25^ Unfortunately, the FDA has reported that the use of MTX is associated with severe toxicities, such as bone marrow suppression, aplastic anemia, gastrointestinal toxicity, and opportunistic infections, which can lead to discontinuation of therapy and even be life‐threatening.^26^, ^27^, ^28^, ^29^, ^30^, ^31^ Another strategy involved the utilization of biosimilars. ABP 501 (Adalimumab‐atto) was approved as the first Adalimumab biosimilar by the FDA.^32^ A study by Cohen et al. indicated that the clinical efficacy, safety, and immunogenicity profiles of ABP 501 were highly similar to those of the reference adalimumab product in patients with moderate to severe RA.^33^ However, there was still a concern that the long‐term use of biosimilars could potentially induce the formation of anti‐Id Abs, which might impact the efficacy and safety of the biosimilar over extended periods. Therefore, it is important to develop a universal strategy to prevent the interference of anti‐Id Abs in Ab drug treatment without any adverse effects.

**FIGURE 1:**
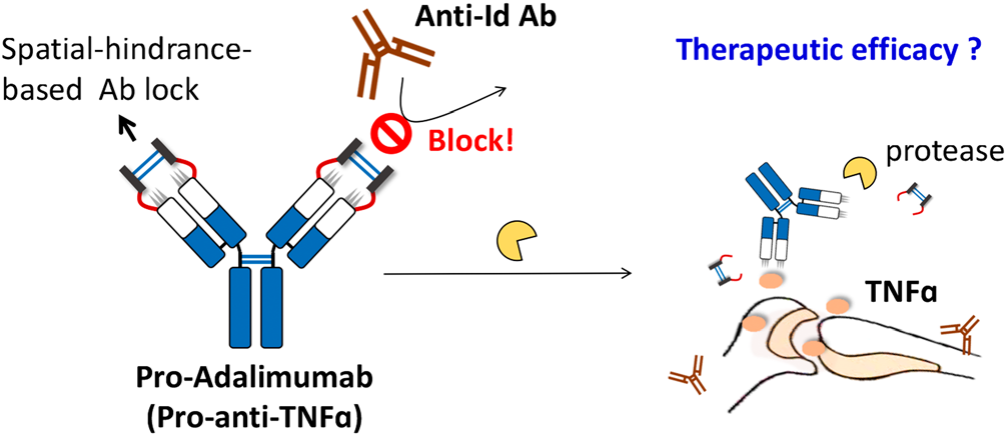
Development of a spatial‐hindrance‐based Ab lock to prevent interference from anti‐Id Ab during Adalimumab treatment.

In this study, to avoid the interference of an anti‐Id Ab, we used autologous hinge regions^34^ as a spatial hindrance‐based Ab lock to cover the CDR binding site of Adalimumab, along with a substrate peptide (MMP‐2/9)^10^, ^35^, ^36^, ^37^, ^38^ that connects the Ab lock to the N‐terminal of the light chain and heavy chain to generate pro‐Adalimumab. Pro‐Adalimumab can prevent the interference of anti‐Id Ab and be selectively activated by protease in disease regions to decrease RA progression. Furthermore, it maintained consistent PK properties in the presence of anti‐Id Ab (Figure 1). We compared the protease‐cleaving and Ag‐blocking abilities of pro‐Adalimumab to Adalimumab by western blot and enzyme‐linked immunosorbent assay (ELISA) and further confirmed the protease‐restoration ability of pro‐Adalimumab with MMP‐2/9 treatment. Furthermore, we assessed the impact of anti‐Adalimumab Id Ab on the ability of pro‐Adalimumab and Adalimumab to bind TNFɑ with or without MMP‐2/9 treatment. In addition, we also determined the interference of anti‐Adalimumab Id Ab on the PK properties and therapeutic efficacy of pro‐Adalimumab and Adalimumab in vivo. In summary, we developed the spatial hindrance‐based Ab lock to prevent the interference of anti‐Id Ab and enhance efficacy. Such a strategy has potential for use in patients who have developed anti‐Id Ab and provide the second‐line drug in the clinic.

To avoid interference from anti‐Id Abs, we used an autologous hinge region as a spatial‐hindrance‐based Ab lock and connected it to the N‐terminal of the light chain and heavy chain via substrate peptides (MMP‐2/9) to cover the CDR binding site of Adalimumab to generate pro‐Adalimumab. Pro‐Adalimumab can block the neutralization effect of anti‐Id Abs and be selectively activated in the disease area. The spatial‐hindrance‐based Ab lock can be completely removed from pro‐Adalimumab after protease cleavage at the RA region, thus restoring the TNFɑ‐binding ability of Adalimumab and enhancing the efficacy of RA therapy in the presence of anti‐Id Ab. Moreover, pro‐Adalimumab showed similar pharmacokinetics to Adalimumab.

## 2 METHODS

### 2.1 Cell and animals

Expi293F cells (Thermo Fisher Scientific, Waltham, MA, USA) were cultured in Expi293 Expression Medium at 37°C in a humidified atmosphere of 8% CO_2_. Female C57BL/6 mice were obtained from the National Laboratory Animal Center in Taipei, Taiwan. Female C57BL/6 mice exhibit reduced hormonal fluctuations and stronger immune responses, making them more consistent for immune experiments than male mice.^39^, ^40^ The protocol for male human TNFɑ transgenic mice followed the methods outlined in our previous study.^34^ Male mice demonstrated earlier onset and greater severity in the evaluation of the arthritis model.^41^ Animal experiments were approved by the Institutional Animal Care and Use Committee (IACUC approval number 106271, Kaohsiung Medical University, Kaohsiung, Taiwan) and carried out in accordance with institutional guidelines.

### 2.2 Construction and production of pro‐Adalimumab

The complementary DNA coding for the heavy and light chains of Adalimumab was cloned through assembly PCR. Human IgG1 hinge sequences were obtained from the National Center for Biotechnology Information. The hinge‐encoding sequences, HRGDS linker, and MMP‐2/9 substrate‐encoding sequences (GPLGVR)^34^, ^42^ were introduced upstream of the light chain and heavy chain of Adalimumab to generate pro‐Adalimumab. Adalimumab or pro‐Adalimumab was produced through the Expi293 Expression System (Thermo Fisher Scientific, Waltham, MA, USA). The protein was purified by protein A‐sepharose (GE Healthcare, Milwaukee, WI, USA), and their molecular weights were found using SDS‐PAGE.

### 2.3 The antigen binding ability of pro‐Adalimumab with or without MMP‐2/9

To determine the binding kinetics (EC_50_) of pro‐Adalimumab and Adalimumab, 96‐well plates were coated with human TNFɑ (0.3 μg/mL, R&D systems, Minneapolis, MN, USA) and blocked with 5% skim milk. Pro‐Adalimumab or Adalimumab was incubated with or without 20 μg/mL of MMP‐2/9 (type IV collagenase, Sigma‐Aldrich, St. Louis, MO, USA) in DMEM/0.05% BSA for 1 h at 37°C before terminating the reaction with bovine calf serum (BCS). The samples were added to the plates at given concentrations (200–0.0001 nM) for 1 h at RT. After washing, the wells were incubated with horseradish peroxidase (HRP)‐goat anti‐human IgG Fcγ Ab for 1 h at RT, and detection was performed by adding ABTS containing 30% H_2_O_2_. The binding ability was quantified through absorbance detection at 405 nm. Pro‐Adalimumab and Adalimumab were evaluated by western blot using HRP anti‐hIgG Fab Ab and HRP anti‐hIgG Fc Ab (Jackson ImmunoResearch Laboratories, West Grove, PA, USA).

### 2.4 The binding ability of anti‐idiotypic Abs to pro‐Abs

To assess the binding ability of anti‐idiotypic antibodies (anti‐Id Abs) to pro‐Abs, ELISA assays were performed for pro‐Adalimumab, pro‐Infliximab, and pro‐Nivolumab. For pro‐Adalimumab, anti‐Adalimumab Id Ab (0.2 μg/mL, HCA204G, Bio‐Rad Laboratories, Redmond, WA, USA) was coated onto 96‐well plates and blocked with 5% skim milk. Pro‐Adalimumab or Adalimumab was added at given concentrations (0.03125–10 μg/mL) and incubated for 1 h at room temperature (RT). After washing, the wells were incubated with biotin‐conjugated anti‐myc Ab for 1 h at RT, followed by HRP‐Streptavidin and ABTS containing 30% H₂O₂ for detection. Absorbance was measured at 405 nm. For pro‐Infliximab,^34^ anti‐Infliximab Id Ab (0.3 μg/mL, HCA202, Bio‐Rad Laboratories, Redmond, WA, USA) was coated onto 96‐well plates and blocked with 5% skim milk. Pro‐Infliximab or Infliximab was added at given concentrations and incubated for 1 h at RT. After washing, the wells were incubated with HRP‐conjugated anti‐Id whole Ab (HCA203P, Bio‐Rad Laboratories, Redmond, WA, USA) for 1 h at RT, followed by ABTS containing 30% H₂O₂ for detection. Absorbance was measured at 405 nm. For pro‐Nivolumab, 3 μg/mL pro‐Nivolumab or Nivolumab was coated onto 96‐well plates and blocked with 5% skim milk. Anti‐Nivolumab Id Ab (6G5) biotin (A01848, GenScript USA, Inc.) was added at given concentrations (500–6.2 ng/mL) and incubated for 1 h at RT. Detection was performed using HRP‐Streptavidin and ABTS containing 30% H₂O₂, with absorbance measured at 405 nm.

### 2.5 The protease‐restoring ability of pro‐Adalimumab in the presence of anti‐idiotypic Abs

Ninety‐six‐well plates were coated with 5 μg/mL or 5.5 μg/mL of Pro‐Adalimumab or Adalimumab and blocked with 5% skim milk. Anti‐Id Adalimumab Ab was incubated with different concentrations (8–1000 ng/mL) and unbound Ab was removed after washing. Each sample was then incubated with or without 20 μg/mL of MMP‐2/9 for 1 h at RT. After washing, the wells were incubated with Biotinylated TNFɑ (ACROBiosystems, USA) for 1 h at RT and sequentially incubated with HRP‐conjugated streptavidin. Detection was performed by adding ABTS containing 30% H_2_O_2_, and the binding ability was quantified through absorbance detection at 405 nm.

### 2.6 The PK properties of pro‐Adalimumab in the presence of anti‐idiotypic Abs in vivo

To validate the experimental rationale, we referenced the typical concentration of anti‐Id Abs observed at the disease site in human patients to determine the appropriate amount of anti‐Id Abs to inject into the mice. Bartelds et al. reported that among 272 patients who received 40 mg of adalimumab subcutaneously every other week, 28% developed anti‐adalimumab Abs during a 156‐week follow‐up.^20^ The titers of anti‐adalimumab Abs ranged from 13 to 17 AU/mL (equivalent to 156–204 ng/mL, with 1 AU ≈ 12 ng). In our experiment, six‐week‐old C57BL/6 mice were randomly divided into groups of two mice per group. Each mouse was intravenously injected with either 10 μg of pro‐Adalimumab or 10.7 μg of Adalimumab. At 90 min post‐inoculation with the Ab, mice were injected with or without anti‐Adalimumab Id Ab at doses corresponding to Ab titers ranging from 5.1 to 7.6 μg/mL. Whole blood was collected via the tail vein at different time points. Ab concentrations in mouse plasma were detected by ELISA. We coated 96‐well plates with 2.5 μg/mL anti‐his Ab and blocked with 5% skim milk. Plasma containing pro‐Adalimumab or Adalimumab at dilutions of 100‐ and 300‐fold, as well as serially diluted Adalimumab standards (in duplicate), were added to the plates for 1 h at RT. After washing, the wells were incubated with 2 μg/mL biotin‐conjugated anti‐myc Ab for 1 h at RT. Subsequently, the reaction was incubated with HRP‐Streptavidin for 1 h at RT, and detection was performed by the addition of ABTS containing 30% H_2_O_2_. The binding ability was quantified through absorbance detection at 405 nm.

### 2.7 Therapeutic efficacy of pro‐Adalimumab in the presence of anti‐idiotypic Abs in an RA mouse model

Six‐week‐old male human TNFɑ transgenic mice were randomly divided into groups of six mice per group and intraperitoneally injected with 2 mg/kg Adalimumab or 2.2 mg/kg pro‐Adalimumab, followed by injections with or without 2 mg/kg anti‐idiotypic Adalimumab Ab and saline 30 min after the initial antibody injection. All mice received doses twice weekly for 5 weeks, and the therapeutic efficacy was monitored weekly by evaluating clinical scores and body weight, using a previously described scoring system.^26^, ^43^ After 6 weeks of treatment, all the mice were sacrificed, and the hind ankle joints were removed for histology. The paws were fixed with formalin buffer for HE staining.

### 2.8 Statistical analysis

The data are presented as mean ± SEM or SD. Statistical significance between groups was compared by analyzing the concentration of pro‐Adalimumab and Adalimumab in the blood specimens using ANOVA. The PK properties of pro‐Adalimumab and Adalimumab treated with or without anti‐Adalimumab Id Ab in mice were calculated by one‐phase decay exponential regression. The statistical analysis was performed using GraphPad Prism v.6, and the results were considered significant at a *p* value of less than 0.05.

## 3 RESULTS

### 3.1 The spatial‐hindrance‐based Ab lock can efficiently reduce the antigen‐binding ability of pro‐Adalimumab

**FIGURE 2:**
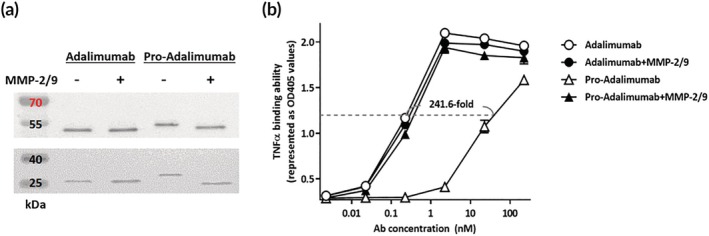
Ag‐binding and protease restoration through pro‐Adalimumab.

To examine whether the spatial‐hindrance‐based Ab locks could reduce the Ag binding ability of pro‐Adalimumab and restore the activity of Adalimumab after MMP‐2/9 treatment, we evaluated the protease‐cleavability of pro‐Adalimumab with or without incubation with MMP‐2/9 using western blot analysis, and further confirmed the Ag blocking ability and protease‐restoration ability of the pro‐Adalimumab by hTNFɑ‐based ELISA. As shown in Figure 2a, the molecular weight (MW) of the light chain (LC) and heavy chain (HC) of Adalimumab did not change with MMP‐2/9 treatment. In contrast, the MW of LC and HC of pro‐Adalimumab decreased from 26.4 to 23.43 kDa and 58.84 to 49.3 kDa, respectively, indicating pro‐Adalimumab can be removed entirely by MMP‐2/9 treatment. As shown in Figure 2b, the Ag‐binding half maximal effective concentrations (EC_50_) of pro‐Adalimumab and Adalimumab were 74.91 and 0.31 nM, showing that pro‐Adalimumab had a 241.6‐fold enhanced blocking ability compared with Adalimumab. After MMP‐2/9 treatment for 2 h, Ag binding ability was completely restored by pro‐Adalimumab to a level similar to Adalimumab. These results show that the spatial‐hindrance‐based Ab lock can efficiently block the Ag‐binding ability of pro‐Adalimumab. After MMP‐2/9 treatment, the Ab lock can be completely cleaved from pro‐Adalimumab, thus restoring the binding ability to that of Adalimumab.

Adalimumab and pro‐Adalimumab were pre‐incubated with or without MMP‐2/9. Western blot shows the MW of LC and HC on Adalimumab or pro‐Adalimumab, which were detected by anti‐human IgG Fcγ conjugated with horseradish peroxidase (HRP). (a) The Abs were added to 96‐well plates coated with hTNFɑ. The binding ability of Adalimumab (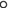), pro‐Adalimumab (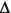) and MMP‐2/9‐activated Adalimumab (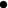), pro‐Adalimumab (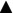) were assessed by hTNFɑ‐based ELISA. (b) The values are mean ± SD. Error bar: mean of two repeated measurements. Ab, antibody; LC, light chain; HC, heavy chain; MMP, matrix metalloproteinase; MW, molecular weight; TNFɑ, tumor necrosis factor ɑ.

### 3.2 The spatial‐hindrance‐based Ab inhibits the binding ability of anti‐Adalimumab Id Ab

**FIGURE 3:**
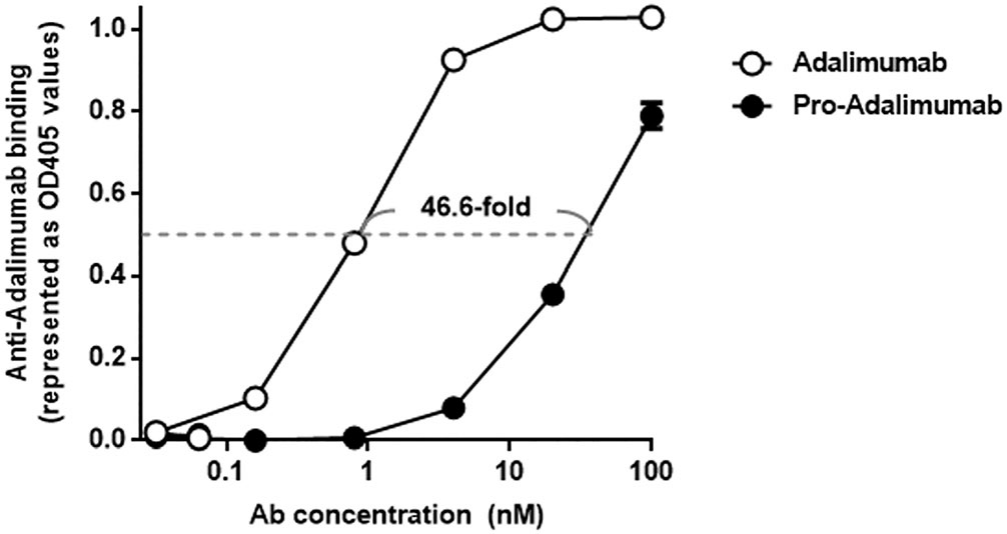
Comparison of the binding ability of anti‐Adalimumab Id Ab to pro‐Adalimumab and Adalimumab.

To examine the efficiency of the spatial‐hindrance‐based Ab lock in preventing the binding of the anti‐Id Ab, we coated the ELISA plate with anti‐Adalimumab Id Ab (clone: AbD18655_hIgG1). Then we incubated it with the different indicated concentrations (0.03125–10 μg/mL) of pro‐Adalimumab and Adalimumab to analyze the binding ability of anti‐Adalimumab Id Ab by using anti‐Myc‐biotin Ab and HRP‐Streptavidin. As shown in Figure 3, the binding ability of pro‐Adalimumab to anti‐Adalimumab Id Ab was weaker than that of Adalimumab. The anti‐Adalimumab Id Ab‐binding EC_50_ of pro‐Adalimumab and Adalimumab was 49.87 and 1.07 nM, respectively, indicating that pro‐Adalimumab retained a 46.6‐fold higher blocking ability against anti‐Adalimumab Id Ab compared to Adalimumab. This result suggests that the spatial‐hindrance‐based Ab lock can efficiently prevent the binding of the anti‐Id Ab.

The 96‐well plate coated with anti‐Adalimumab Id Ab was incubated with different concentrations of Adalimumab or pro‐Adalimumab. After removing unbound Adalimumab or pro‐Adalimumab, we added anti‐Myc‐biotin Ab and HRP‐Streptavidin to analyze the binding ability of Adalimumab (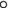) and pro‐Adalimumab (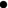) to anti‐Adalimumab Id Ab. The values are mean ± SD. Error bar: mean of two repeated measurements.

### 3.3 The spatial‐hindrance‐based Ab lock can inhibit the binding ability of various anti‐Id Abs

To further prove whether the Ab lock would prevent the binding ability of anti‐Id Abs for various clinical Abs, we employed the same strategy as described above to generate pro‐Abs, pro‐Infliximab (anti‐TNFɑ Ab), and pro‐Nivolumab (anti‐programmed cell death protein 1 (PD‐1) Ab). The binding ability of the anti‐Id Ab to the original Ab and pro‐Ab was analyzed by performing individual ELISAs. The results demonstrated that the binding EC_50_ of anti‐Infliximab Id Ab to Infliximab and pro‐Infliximab was 4.91 and 254.40 nM (51.8‐fold blocking ability), respectively. For anti‐Nivolumab Id Ab to Nivolumab and pro‐Nivolumab, the EC_50_ values were 18.21 and 358.16 nM (19.7‐fold blocking ability), respectively (Table 1). These results suggest that the spatial‐hindrance‐based Ab lock may be widely applicable and can prevent the binding ability of anti‐Id Abs to different Ab drugs.

**TABLE 1:** The Ab lock can be widely applied to clinical Ab drugs and prevent the binding ability of anti‐Id Abs.

Type of anti‐Id Abs	EC‐50 (nM)		Fold
	Original Ab	Pro‐Ab	
Anti‐Adalimumab Id Ab	1.07	49.87	46.6
Anti‐Infliximab Id Ab	4.91	254.40	51.8
Anti‐Nivolumab Id Ab	18.21	358.16	19.7

*Note*: The Ab lock can inhibit the binding abilities of various types of anti‐Id Abs to different pro‐Abs. EC_50_ represents the half‐maximal effective concentration. Blocking fold is calculated as pro‐Ab EC_50_/original Ab EC_50_.

### 3.4 The spatial‐hindrance‐based Ab lock prevents anti‐Adalimumab Id Ab neutralization, preserving the protease‐restoration ability of pro‐Adalimumab

**FIGURE 4:**
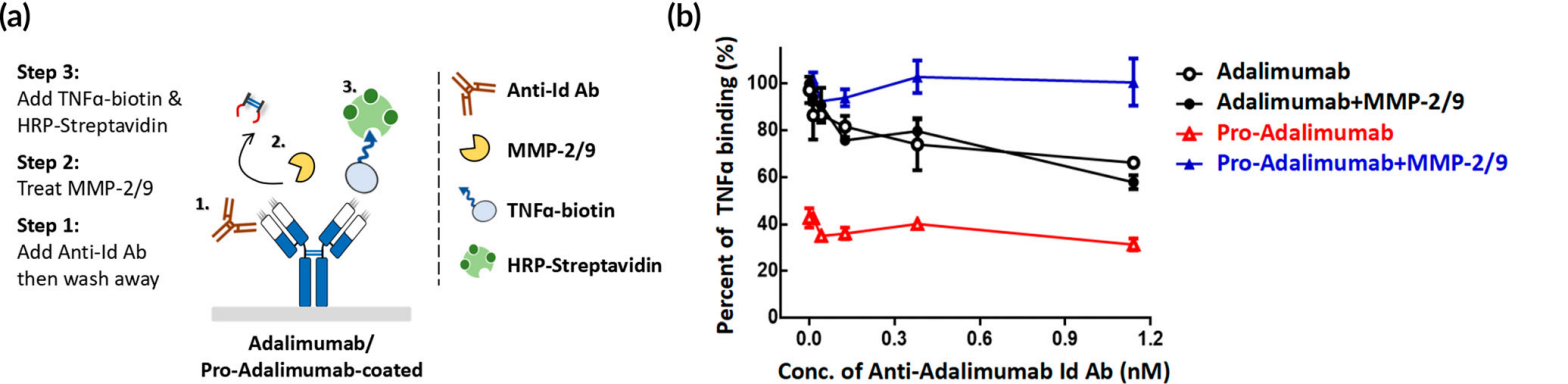
The Ag‐binding and protease‐restoration ability of pro‐Adalimumab in the presence of anti‐Adalimumab Id Ab.

To understand whether pro‐Adalimumab can maintain protease‐restoration ability, with or without MMP‐2/9 treatment, the pre‐coated Adalimumab or pro‐Adalimumab were incubated with different indicated concentrations (0–1.14 nM) of anti‐Adalimumab Id Ab. Following protease treatment, TNFɑ‐biotin and HRP‐Streptavidin were added to analyze the protease‐restoration ability of pro‐Adalimumab (Figure 4a). As shown in Figure 4b, the TNFɑ‐binding ability of Adalimumab declined from 100% to 66.2% after anti‐Adalimumab Id Ab treatment. In contrast, the TNFɑ‐binding ability of pro‐Adalimumab, with or without MMP‐2/9 treatment, remained unaffected by anti‐Adalimumab Id Ab treatment. In addition, pro‐Adalimumab pre‐treated with anti‐Adalimumab Id Ab could restore 100% Ag‐binding ability after MMP‐2/9 treatment. The result indicated that pro‐Adalimumab was not affected by the presence of anti‐Id Ab and could completely restore the Ag binding ability after MMP‐2/9 treatment.

Adalimumab or pro‐Adalimumab was coated onto 96‐well plates, followed by incubation with anti‐Adalimumab Id Ab over a range of serial dilutions. After washing away the unbound anti‐Adalimumab Id Ab, MMP‐2/9 was added to the plates, and the TNFɑ‐binding ability of the Abs was analyzed by adding TNFɑ‐biotin and HRP‐Streptavidin. Adalimumab (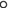), Adalimumab+MMP‐2/9 (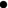), pro‐Adalimumab (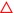), pro‐Adalimumab+MMP‐2/9 (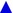). The values are mean ± SD. Error bar: mean of two repeated measurements.

### 3.5 Pro‐Adalimumab can prevent the neutralizing effect of anti‐Adalimumab Id Ab and prolong its half‐life in vivo

**FIGURE 5:**
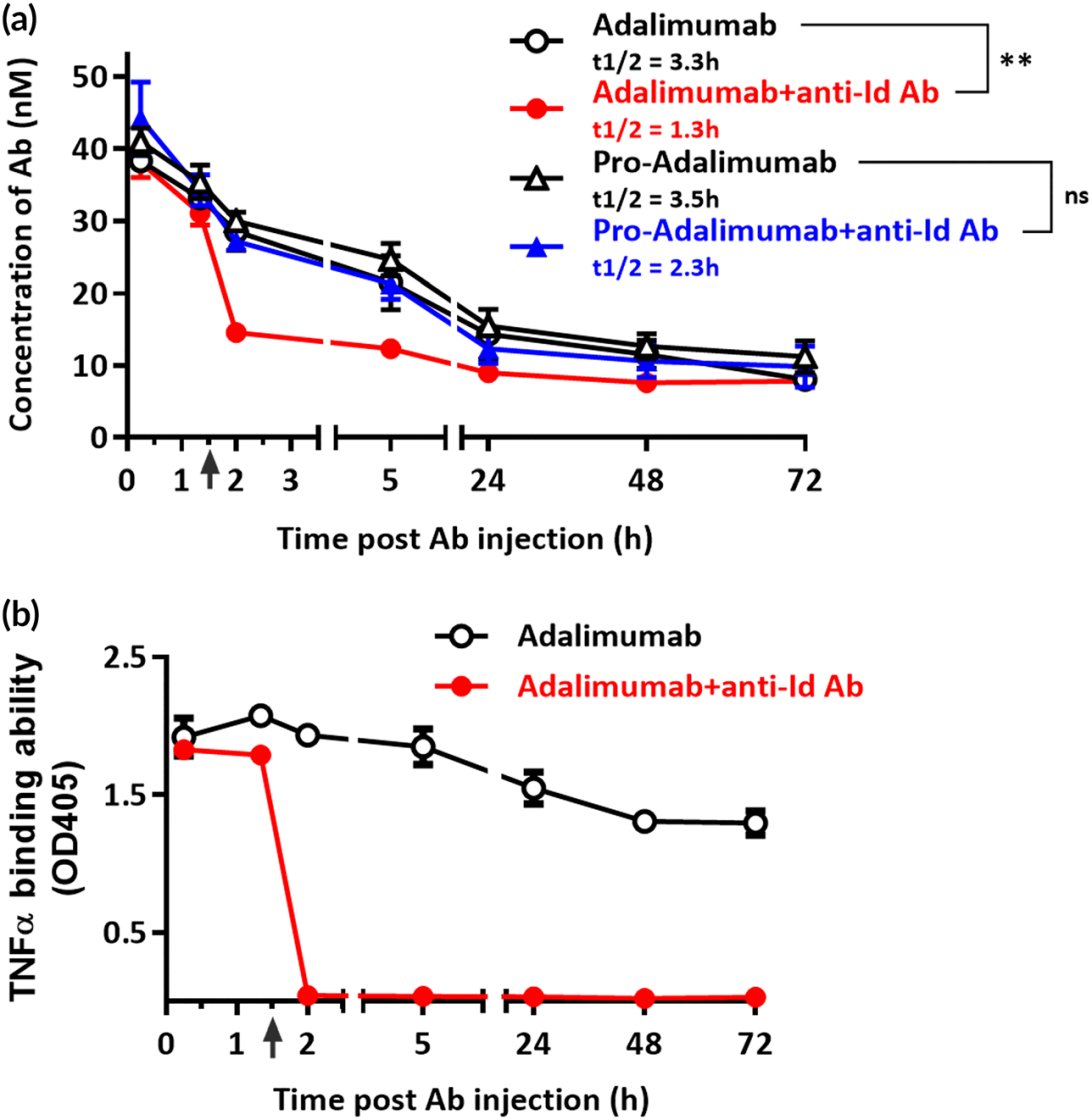
The PK properties of pro‐Adalimumab in the presence of anti‐Adalimumab Id Ab.

We further evaluated whether the spatial‐hindrance‐based Ab lock could efficiently prevent the neutralization of the anti‐Id Abs and reduce clearance by measuring the PK properties of pro‐Adalimumab. The results indicated that pro‐Adalimumab was not affected by the presence of anti‐Id Ab and could completely restore the Ag binding ability after MMP‐2/9 treatment in vivo. C57BL/6 mice were intravenously injected with pro‐Adalimumab or Adalimumab and subsequently injected with or without anti‐Adalimumab Id Abs at 90 min post‐injection of the Ab. Ab concentration in the presence of anti‐Id Ab was examined by sandwich ELISA. As shown in Figure 5a, the concentration of Adalimumab with anti‐id Ab in the blood decreased by 49% in mice compared with Adalimumab‐treated mice without anti‐Adalimumab Id Ab, but the concentration of pro‐Adalimumab with anti‐Adalimumab Id Ab only decreased by 9.4% in mice compared with pro‐Adalimumab‐treated mice without anti‐Adalimumab Id Ab after 120 min following the Ab injection. The half‐life of Adalimumab and pro‐Adalimumab was 3.3 and 3.5 h, respectively, compared to 1.3 and 2.3 h for Adalimumab with anti‐Adalimumab Id Ab and pro‐Adalimumab with anti‐Adalimumab Id Ab, respectively. Among the above administrations, the concentration of pro‐Adalimumab was not significantly different between anti‐Adalimumab Id Ab treatment and no treatment (*p* = 0.6712). In contrast, Adalimumab showed a significant difference between anti‐Adalimumab Id Ab treatment and no treatment (*p* < 0.01), suggesting that the Ab lock of pro‐Adalimumab prevented the interference of anti‐Adalimumab Id Ab in mice compared with Adalimumab. Additionally, we confirmed whether the anti‐Adalimumab Id Ab could cause Adalimumab to lose its binding function in mice. As shown in Figure 5b, the TNFɑ‐binding function of Adalimumab in Adalimumab + anti‐Adalimumab Id Ab treated mice was decreased by 97.9% compared with Adalimumab‐treated mice 120 min after the Ab injection, indicating that an anti‐Adalimumab Id Ab can impair the binding ability of Adalimumab. These results suggest that the spatial‐hindrance‐based Ab lock can prevent the neutralizing effect of the anti‐Adalimumab Id Ab, thus prolonging the half‐life of Adalimumab in vivo.

Adalimumab and pro‐Adalimumab were intravenously injected into C57BL/6 mice (*n* = 2 per group). After 90 min, the mice were intravenously injected with anti‐Adalimumab Id Ab. Blood was harvested at different times, and the concentrations of (A) Abs and (B) TNFɑ‐binding ability were analyzed by ELISA. The gray arrow represents the period of anti‐Adalimumab Id Ab administration. Adalimumab (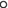), Adalimumab + anti‐Id Ab (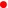) pro‐Adalimumab (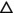), pro‐Adalimumab + anti‐Id Ab (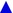). Data are represented as mean ± SEM. Statistical analysis was performed using ANOVA. Half‐life values were calculated using one‐phase decay exponential regression. **, *p* < 0.01; ns, not significant.

### 3.6 Pro‐Adalimumab can prevent anti‐Adalimumab Id Ab interference, efficiently reducing RA progression

**FIGURE 6:**
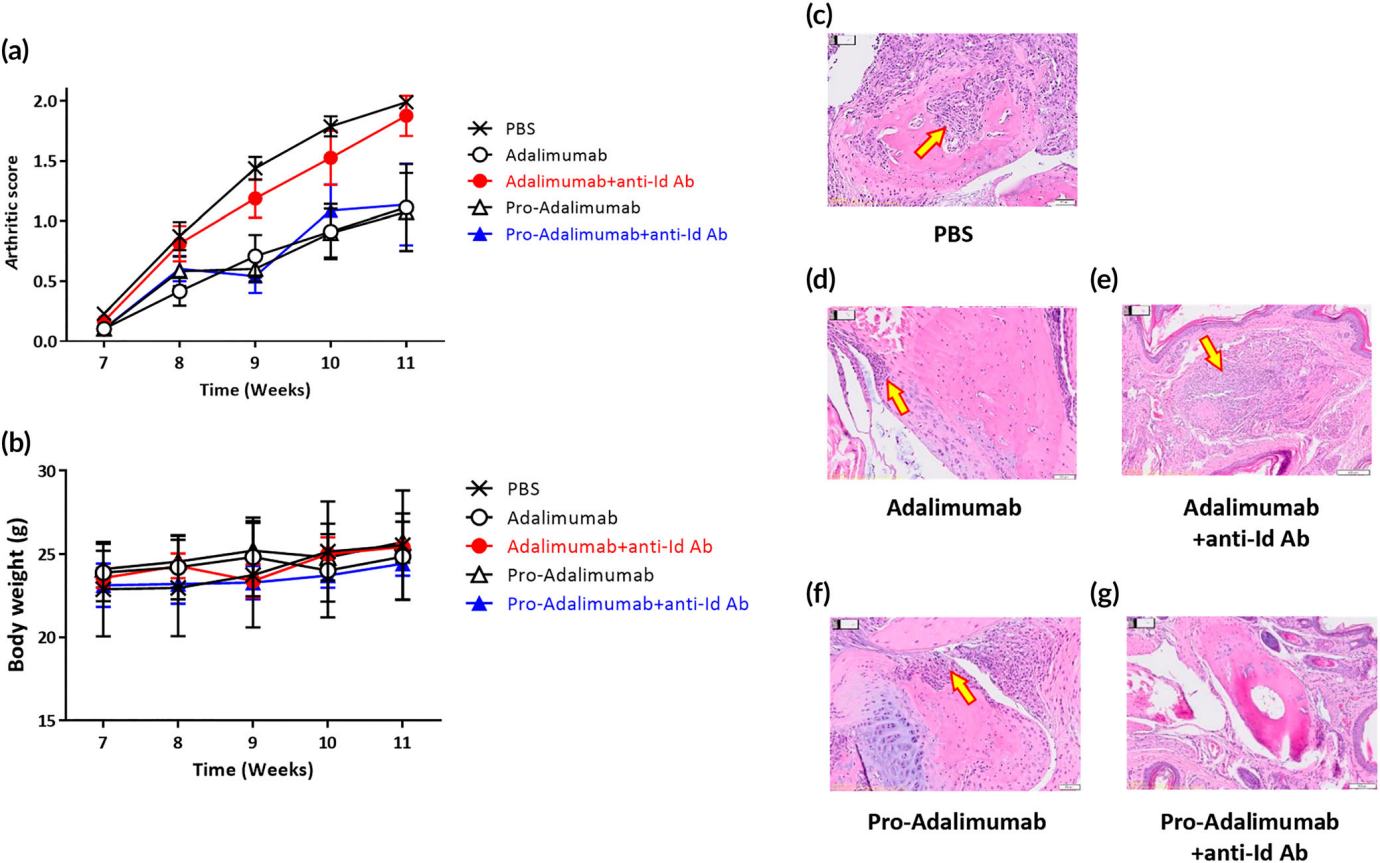
The therapeutic efficacy of pro‐Adalimumab in human TNFɑ transgenic mice.

We further confirmed whether pro‐Adalimumab could maintain therapeutic efficacy in the presence of anti‐Adalimumab Id Ab. Mice (*n* = 6) were intraperitoneally injected with pro‐Adalimumab or Adalimumab and further injected with or without anti‐Adalimumab Id Ab. As shown in Figure 6a, the clinical scores for pro‐Adalimumab‐ and Adalimumab‐treated mice were decreased compared with control mice treated with PBS, indicating both pro‐Adalimumab and Adalimumab were effective in reducing RA progression. However, Adalimumab‐treated mice with anti‐Adalimumab Id Ab exhibited severe arthritis compared with the Adalimumab‐treated mice without anti‐Adalimumab Id Ab, indicating that anti‐Adalimumab Id Ab interfered with Adalimumab and reduced its efficacy for RA therapy. In contrast, the administration of anti‐Adalimumab Id Ab injection did not affect the score of pro‐Adalimumab‐treated mice, demonstrating that pro‐Adalimumab can prevent the interference of anti‐Adalimumab Id Ab in vivo, thereby reducing RA progression. As shown in Figure 6b, the Ab‐treated mice with or without anti‐Adalimumab Id Ab showed similar body weights. Moreover, joint damage in the paws was evaluated by histopathologic analysis through hematoxylin–eosin (HE) staining. PBS‐treated mice showed a severe inflammatory response due to the infiltration of macrophages, plasma cells, and a few lymphocytes (Figure 6c). The pathological images of the Adalimumab‐ and pro‐Adalimumab‐treated groups clearly showed a decreased inflammatory area compared to the PBS‐treated group (Figure 6d,f). Notably, the Adalimumab‐treated group with anti‐Adalimumab Id Abs exhibited a more severe inflammatory response, indicated by an increased infiltration area, than the pro‐Adalimumab‐treated group with anti‐Adalimumab Id Abs (Figure 6e,g). Taken together, in hTNFɑ transgenic mice, we confirmed that pro‐Adalimumab can reduce the arthritis level and pathological progression in the presence of anti‐Adalimumab Id Ab, proving that the spatial‐hindrance‐based Ab lock can efficiently inhibit the interference of anti‐Id Ab.

Adalimumab (2 mg/kg) and pro‐Adalimumab (2.2 mg/kg) were repeatedly injected intraperitoneally into human TNFɑ transgenic mice (*n* = 6) twice a week for 5 weeks and further injected with or without anti‐Adalimumab Id Ab at 30 min post‐inoculation of the Abs. The arthritic score and body weight were recorded during the Ab injection process. The arthritic score (A) and body weight (B) of each group were monitored once every week to measure the therapeutic efficacy. PBS (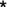), Adalimumab (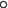), Adalimumab + anti‐Adalimumab Id Ab (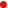) pro‐Adalimumab (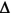), pro‐Adalimumab + anti‐Adalimumab Id Ab (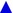). Data are represented as mean ± SEM. Error bar: mean of six repeated measurements.

## 4 DISCUSSION

In this study, we successfully used a spatial‐hindrance‐based and autologous Ab lock^34^ to develop pro‐Adalimumab, which efficiently prevented the neutralization effect of anti‐Id Abs during RA treatment. Our results demonstrate that pro‐Adalimumab has PK properties and therapeutic efficacy similar to Adalimumab, whether or not the anti‐Adalimumab Id Ab is present in the blood. This suggests that the Ab lock can circumvent the interference of anti‐Adalimumab Id Ab in vivo. The immunogenic production of anti‐Id Abs due to long‐term or repeated administration of Ab drugs constitutes a serious clinical issue, potentially decreasing the efficacy of, or interrupting therapies.^44^, ^45^ Some clinical Ab drugs have also been reported to elicit an anti‐Id Ab response after treatment, such as the targeted cancer drug Campath (anti‐CD52 Ab) and the immunosuppressant drug Remicade (anti‐TNFɑ Ab).^15^, ^46^, ^47^ We successfully developed an autologous Ab lock (hinge) to avoid interference from anti‐Id Abs. Previously, we also demonstrated that the Ab lock can enhance the selectivity and mitigate on‐target toxicities of Remicade.^34^ The spatial‐hindrance‐based Ab lock can easily transform any Ab drugs into pro‐Abs, ensuring safety and offering potential second‐line treatment for patients who have developed anti‐Id Abs.

Our results proved that pro‐Adalimumab can be activated by MMP‐2/9 and achieve efficacy in vivo. We hypothesize that in the disease region, activated pro‐Adalimumab can target TNFɑ rather than being neutralized by anti‐Adalimumab Id Ab, based on the following presumptions: (1) pro‐Adalimumab is masked by a spatial‐hindrance‐based Ab lock in the blood circulation, preventing systemic anti‐Adalimumab Id Ab interference with pro‐Adalimumab; (2) soluble TNFɑ competes most efficiently with Adalimumab, with a dissociation equilibrium constant (*K*_D_) value of 30 pM, whereas anti‐Adalimumab Id Ab is slightly less active in the disease region (*K*_D_ values of 60 pM)^48^, ^49^; (3) patients suffering from RA present with a high concentration of TNFɑ at the site of inflammation and in the circulation. A statistics study showed that serum TNFα levels were remarkably elevated in the RA patients (17.9 ± 3.6 pg/mL) compared to a healthy group (5.5 ± 3.3 pg/mL).^50^ Therefore, anti‐Adalimumab Id Ab did not easily neutralize the activated pro‐Adalimumab, as expected in the disease site. Taking these results together, we demonstrated that pro‐Adalimumab could regain its activity to neutralize TNFɑ without interference from anti‐Adalimumab Id Ab in the disease region, further reducing the RA progression.

Development of a pro‐Abs with a low immunogenic Ab lock is important to avoid producing unwanted immunogenicity. However, pro‐Abs, which use exogenous or recombinant structures as masking domains, still carry immunogenic risks. For example, Desnoyers et al. screened a bacterial display‐associated binding peptide as the masking domain to develop the pro‐ɑEGFR Ab, which was connected with a substrate peptide of urokinase‐type plasminogen activator (uPA).^51^ The exogenous binding peptide posed a risk of eliciting undesirable immune responses. Trang et al. used the leucine zipper coiled‐coil domain as a masking domain to block the CDRs of ɑCD20, ɑHER2, and ɑCD3 Ab by using MMP‐2 or ‐9 substrate linkers to develop pro‐Abs.^52^ However, using a complex helix structure‐based coiled‐coil domain introduces potential immunogenic risks.^53^ Our previous studies compared the immunogenicity of coiled‐coil masking and autologous hinge masking pro‐infliximab (anti‐TNFɑ Ab) in vivo. The results indicated that the autologous hinge masking pro‐infliximab did not induce the anti‐Ab lock Ab, but the coiled‐coil masking pro‐infliximab elicited an immune response and accelerated the Ab clearance in blood, suggesting that the autologous hinge possesses the characteristics of low immunogenicity [data not shown]. We have demonstrated that the autologous hinge exhibits low immunogenicity and efficiently avoids interference from anti‐Id Ab to Ab drugs in the systemic circulation. However, additional research is required to definitively establish whether the Ab lock system exhibits equivalent or reduced immunogenicity compared to biosimilars, which would validate its potential as a clinical alternative.

It is important to entirely block the complementarity‐determining region (CDR) loops of Ab drugs to prevent the binding of anti‐Id Abs, but there is limited information about where the anti‐Id Abs exhibit specific dominant positions on the CDRs due to the high specificity of each anti‐Id Ab. van Schouwenburg and colleagues generated 16 anti‐adalimumab Abs derived from different precursor B cells demonstrating that each Ab contains multiple epitopes involved in the CDRs of both the heavy and light chains. This suggests that anti‐adalimumab Abs bind to distinct epitopes on adalimumab.^54^ To effectively mask the majority of binding sites on Ab drugs and prevent interference from anti‐Id Ab, we developed a method using structure‐based computational simulation to predict the cover rate of the Ab lock and select the suitable linkers for different Ab drugs.^55^ The cover rate was determined by a homemade program that analyzes the trajectories and calculates the frequency if any atom of hinge, linker, or substrate is above 120° and 4 Å of any atom of CDR amino acids. Through computational simulation, we can accurately predict the trajectory of the Ab lock for any Ab drugs and customize the optimal pro‐Ab to prevent binding by anti‐Id antibodies. Although our computational method offers valuable insights into the potential cover rate of the Ab lock, it is not sufficient on its own to prove the blocking ability of pro‐Adalimumab against multiple anti‐Adalimumab antibodies. Empirical testing will be essential to validate these computational predictions and to ensure that pro‐Adalimumab can effectively neutralize a range of anti‐Id antibodies.

## 5 CONCLUSION

We have demonstrated that an autologous and spatial‐hindrance‐based Ab lock^34^ can efficiently prevent interference from anti‐Adalimumab Id Ab, thus maintaining the PK properties similar to the original drug and reducing the RA progression in the presence of anti‐Adalimumab Id Abs. The Ab lock has the following advantages: (1) the spatial‐hindrance‐based Ab lock can be easily applied to all current Ab drugs; (2) it can enhance Ab safety^34^ and thus solve the anti‐Id Ab dilemma; (3) the computational platform can assist in selecting a suitable Ab lock to simulate the spatial‐hindrance‐based Ab lock for any Ab drugs; (4) it may be suitable to serve as a second‐line drug for patients who develop anti‐Id Abs. We anticipate that the development of pro‐Abs using an Ab lock can solve the issue that leads to anti‐Id Abs and failure in the clinic and have a strong impact on pharmaceutical development.

## AUTHOR CONTRIBUTIONS

**Bo‐Cheng Huang:** Conceptualization; methodology; data curation; formal analysis; investigation; resources; writing – review and editing; writing – original draft; project administration; validation; visualization. **Yu‐Tung Chen:** Conceptualization; data curation; investigation; methodology; resources; validation; writing – original draft; writing – review and editing; project administration; visualization. **Yun‐Chi Lu:** Conceptualization; formal analysis; investigation; supervision; visualization; project administration; resources; validation; methodology. **Kai‐Wen Ho:** Data curation; funding acquisition; formal analysis; methodology; resources; validation; visualization. **Shih‐Ting Hong:** Conceptualization; data curation; methodology; resources; software; visualization. **Tzu‐Yi Liao:** Data curation; funding acquisition; methodology; visualization. **I‐Hsuan Wu:** Data curation; methodology; project administration; resources. **En‐Shuo Liu:** Data curation; funding acquisition; methodology; software. **Jun‐Min Liao:** Formal analysis; software; resources. **Fang‐Ming Chen:** Formal analysis; funding acquisition; supervision. **Chia‐Ching Li:** Methodology; resources. **Chih‐Hung Chuang:** Validation; supervision. **Chiao‐Yun Chen:** Funding acquisition; resources; writing – review and editing; validation; supervision. **Tian‐Lu Cheng:** Conceptualization; methodology; data curation; investigation; formal analysis; supervision; funding acquisition; visualization; resources; writing – original draft; writing – review and editing.

## FUNDING INFORMATION

This work was supported by grants from the Ministry of Science and Technology, Taipei, Taiwan (NSTC 110‐2320‐B‐037‐010‐MY3, NSTC 111‐2124‐M‐037‐001‐MY3, NSTC 111‐2314‐B‐037‐051‐MY3, NSTC 111‐2314‐B‐037‐094‐MY3); the KMU‐KMUH Co‐Project of Key Research (KMUH‐DK(B)110004‐3, KMUH‐DK(B)110006‐1, KMUH‐DK(B)110006‐2, KMUH‐DK(B)111004‐2, KMUH‐DK(B)111001‐3, KMUH‐DK(B)112001‐2) and Research Foundation (KMU‐DK(B)110004‐2, KMU‐DK(B)111004, KMU‐DK(B)111001‐2, KMU‐DK(B)112001‐1, KMU‐DK(B)112001‐3) from Kaohsiung Medical University, Kaohsiung, Taiwan. This work was also supported partially by the Kaohsiung Medical University Research Center Grant (Drug Development and Value Creation Research Center) (KMU‐TC112A03); NTHU‐KMU Joint Research Project (KT112P002).

## CONFLICT OF INTEREST STATEMENT

The authors declare no conflict of interest.

## Data Availability

The data that support the findings of this study are available from the corresponding author upon reasonable request.
